# Erratum to “Social media for research discourse, dissemination, and collaboration in rheumatology”

**DOI:** 10.2478/rir-2023-0017

**Published:** 2023-07-22

**Authors:** Ariella Coler-Reilly, Elizabeth R. Graef, Alfred H.J. Kim, Jean W. Liew, Michael S. Putman, Sebastian E. Sattui, Kristen J. Young, Jeffrey A. Sparks

**Affiliations:** Department of Cell Biology & Physiology, Washington University School of Medicine, St. Louis, MO, USA; Section of Rheumatology, Boston University School of Medicine, Boston, MA, USA; Division of Rheumatology, Medical College of Wisconsin, Milwaukee, WI, USA; Division of Rheumatology and Clinical Immunology, University of Pittsburgh, Pittsburgh, PA, USA; University of Arizona College of Medicine – Phoenix, Phoenix, AZ, USA; Division of Rheumatology, Inflammation, and Immunity, Brigham and Women’s Hospital and Harvard Medical School, Boston, MA, USA

## Connected Content

This corrects the article “Social media for research discourse, dissemination, and collaboration in rheumatology” in volume 3 on page 172.

In the December 2022 issue of Rheumatology and Immunology Research, the paper titled “Social media for research discourse, dissemination, and collaboration in rheumatology” (Rheumatol Immunol Res. 2022;3(4):169-179. doi: 10.2478/rir-2022-0031), was published with some errors:

The sentence “Among 26 rheumatology journal families, 21 (81%) have active Twitter accounts.” should be changed to “Among 28 rheumatology journal families, 24 (86%) have Twitter accounts.” The sentence, “However, only 5 (19%) have a designated social media editor,” should be changed to “However, only 10 (36%) have a designated social media editor.”

In Table 1, some data were presented incorrectly. The corrected Table 1 is shown below. Seminars in Arthritis and Rheumatism was corrected to add @Seminarthrheum and the social media editor. Osteoarthritis and Cartilage was corrected to add the social media editor. BMC Musculoskeletal Disorders / BMC Rheumatology was corrected to change a typographic error in @BMC_series and to add @BioMedCentral. Best Practice & Research in Clinical Rheumatology was corrected to add @best_rheum. The authors note that the use of Twitter for promotion of new publications differs by journals, with some journals posting only selected publications while others promote all new publications.

**Table 1:** Rheumatology journals with Twitter accounts, social media positions in editorial boards and currently utilizing Twitter for promotion of publications

**Journal**	**Twitter Account**	**Use of Twitter for promotion of new publications**	**SoMe position in Editorial Board**
The Lancet Rheumatology	@TheLancetRheum	Yes	No
Nature Reviews in Rheumatology	@NatRevRheumatol	Yes	No
Annals of the Rheumatic Diseases / RMD Open^**^	@ARD_BMJ	Yes	Yes (Advisory board)^*^
Arthritis & Rheumatology/ Arthritis Care & Research / ACR Open Rheumatology^**^	@ACR_Journals	Yes	Yes
Arthritis Research & Therapy	@ArthritisRes	No	No
Seminars in Arthritis and Rheumatism	@Seminarthrheum	Yes	Yes
Therapeutic Advances in Musculoskeletal disease	@TAMuscDis	Yes	Yes
Rheumatology / Rheumatology Advances in Practice^**^	@RheumJnl	Yes	Yes (Advisory board)^*^
The Journal of Rheumatology	@jrheum	Yes	No
Current Opinion in Rheumatology	@CO_Rheumatology	No	No
Clinical and Experimental Rheumatology	@Clinexprheum	No	No
Clinical Rheumatology	@ClinRheumatol	Yes	Yes
Journal of Clinical Rheumatology	@JRheumatology	Yes	No
European Journal of Rheumatology	@EurJRheumatol	No	No
Indian Journal of Rheumatology	@IJRheum	No	Yes (Digital editor)
Reumatismo	@reumatismoJnl	No	No
Reumatologia (Rheumatology)	@ReumatologiaW	No	No
Global Rheumatology	@GlobalRheum	No	No
Rheumatology and Therapy	@RheumatolTher	Yes	No
Advances in Rheumatology	No	NA	NA
Best Practice & Research Clinical Rheumatology	@best_rheum	NA	Yes
BMC Musculoskeletal Disorders / BMC Rheumatology	@BMC_series and @BioMedCentral	No	No
Joint Bone Spine	No	NA	NA
Lupus	@SAGEHealthInfo	Yes	No
Lupus Science and Medicine	@Lupus_SM	Yes	Yes
Osteoarthritis and Cartilage	@OACJournal	Yes	Yes
Rheumatic Disease Clinics of North America	No	NA	NA
Rheumatology and Immunology Research	No	NA	NA

^*^ Advisory boards composed by multiple individuals.

^**^ Affiliated journals.

SoMe, social media; NA, not applicable.

The errors do not change the scientific conclusions of the article. The authors would like to apologize for the errors and any inconvenience caused.

